# Reference values for the adolescent post version of the Postconcussion Symptom Inventory from the German general population

**DOI:** 10.1007/s00431-024-05906-8

**Published:** 2024-12-23

**Authors:** Marina Zeldovich, Leonie Krol, Dagmar Timmermann, Gerard Gioia, Katrin Cunitz, Anna Buchheim, Nicole von Steinbuechel

**Affiliations:** 1 Institute of Psychology, Faculty of Psychology and Sport Science, University of Innsbruck, Innsbruck, Austria; 2 Faculty of Psychotherapy Science, Sigmund Freud University Vienna, Vienna, Austria; 3 Department of Psychology, Clinical Psychology, Experimental Psychopathology, and Psychotherapy, Philipps University of Marburg, Marburg, Germany; 4 Department of Psychosomatic Medicine and Psychotherapy, Division Medical Psychology and Medical Sociology, University Medical Center Goettingen, Goettingen, Germany; 5 Division of Pediatric Neuropsychology, Safe Concussion Outcome Recovery & Education Program, Children’s National Health System, Depts. of Pediatrics and Psychiatry & Behavioral Sciences, George Washington University School of Medicine, Rockville, MA USA

**Keywords:** Reference values, Adolescents, Pediatric traumatic brain injury (pTBI), Postconcussion Symptom Inventory (PCSI)

## Abstract

The present study aims at providing reference values from the general pediatric population for the German version of the 21-item self-report post version of the Postconcussion Symptom Inventory for adolescents aged 13–17 years (PCSI-SR13) following pediatric traumatic brain injury (pTBI). A total of *N* = 950 adolescents completed an adapted version of the PCSI-SR13. Prior to establishing reference values using percentiles, psychometric properties (i.e., reliability and factorial validity) and regression analyses were examined to identify factors contributing to PCSI-SR13 scores. In addition, construct assessment in the general population sample was compared to that in the pTBI sample (*N* = 234) using measurement invariance analyses and direct comparisons at the score levels. The results indicate good reliability (Cronbach’s *α* and McDonald’s *ω* of 0.97 each). The four-factor structure covering physical, emotional, cognitive, and fatigue symptom groups could be replicated with *χ*^2^(183) = 995.96, *p* < 0.001, *χ*^2^/df = 5.44, CFI = 0.99, TLI = 0.98, RMSEA (90% CI) = 0.068 (0.064, 0.073), SRMR = 0.03. With minor restrictions, the assessment of symptoms was comparable between the general population and the pTBI samples. Participants in the pTBI sample reported a significantly higher symptom burden than those in the general population sample. Reference values were provided using the total sample without further stratification.

*Conclusion: *For the German post version of the PCSI-SR13, reference values are now available for direct score comparisons and for drawing conclusions about the clinical relevance of the reported symptoms, while considering the prevalence in a comparable general population without a history of pTBI.

*Trial registration: *The study is retrospectively registered in the German Clinical Trials Register and in the International Clinical Trials Registry Platform (ID DRKS00032854).

**Supplementary Information:**

The online version contains supplementary material available at 10.1007/s00431-024-05906-8.

**Table:** 

**What is Known:**
• *Pediatric traumatic brain injury (pTBI) is a major cause of death and disability, with a wide range of incidence rates and symptoms that interfere with daily functioning and recovery.* • *The Postconcussion Symptom Inventory (PCSI), including recently translated and validated German versions, is a recommended tool for measuring self-reported symptoms in affected children and adolescents.*
**What is New:**
• *This study provides German-specific reference values for the PCSI-SR13, allowing health care professionals to better differentiate between symptoms that may occur in the general adolescent population with and without chronic health conditions and those caused by pTBI.* • *It highlights that adolescents with chronic health conditions but without a history of pTBI often report higher levels of emotional symptoms and fatigue, emphasizing the importance of a nuanced clinical assessment, including medical history.*

## Introduction

Of all types of traumatic injuries, pediatric traumatic brain injury (pTBI) is the one that is most likely to result in death and disability in children and adolescents [1]. The worldwide incidence of pTBI varies widely among countries, with the majority reporting a range of 47 to 280 per 100,000 children [2]. In Germany, pTBI affects about 580 per 100,000 children and adolescents up to 16 years of age annually [3]. The crude incidence rate of pTBI as a primary diagnosis is estimated to be 687 per 100,000 among over 10 million hospital admissions in individuals aged under 18 years between 2014 and 2018 [4]. Given its epidemiology, pTBI poses a challenging problem requiring special attention from clinicians and researchers.

Post-concussion symptoms (PCS) are autonomic (e.g., headache, sensitivity to light and/or noise), vestibular-ocular (e.g., nausea, dizziness), cognitive (e.g., difficulty concentrating), and emotional (e.g., anxiety, irritability) disturbances associated with pTBI [5]. They may occur immediately after the traumatic event and either resolve within the first few days or persist for a longer period of time [6], interfering with daily life [7, 8] and hindering recovery [9].

To assess PCS, clinicians and researchers often refer to the subjective perspective of the affected individual using patient-reported outcome measures (PROMs). One of the PROMs recommended for PCS assessment in pediatric TBI populations [10] is the Postconcussion Symptom Inventory (PCSI) [11]. The PCSI has three age-appropriate self-report forms (for ages 5–7, 8–12, and 13–18, respectively) and a proxy form for ages 5–18. The forms differ in wording and length of the Guttman response scale: three response categories are used for ages up to 12 years, and seven response categories are used for ages 13–18 years and the proxy version. All questionnaires are available in two forms: one for the time before the injury (pre-version) and one for the time after the injury (post version). It is often difficult to obtain valid self-reports of pre-traumatic experiences in children and adolescents, especially if the pTBI occurred in early childhood. To overcome this limitation, only the post version can be administered [11]. Recently, two age-adapted versions, the PCSI-SR8 (for children aged 8–12 years) and the PCSI-SR13 (for adolescents aged 13–18 years), were translated into German, linguistically validated, and psychometrically tested. They have been shown to have good psychometric properties and are comparable to the original English version [12, 13].

In particular, when only the post version of the PCSI is used, it is challenging to assess the clinical relevance of the symptoms reported. In this case, reference values obtained from a comparable general population can be particularly helpful. Reference values reflect the symptom burden in the general population and allow comparisons of the questionnaire score of an individual patient with the corresponding age group from the non-pTBI population. Recently, reference values from the German pediatric population were provided for the PCSI-SR8 [12]. To fill the gap of missing reference values for adolescents, the present study aims to provide these for the PCSI-SR13.

## Materials and methods

### Participants

The data collection was conducted online from March 2022 to April 2022 using the databases of two German-based market research agencies (Dynata, https://www.dynata.com; respondi, https://www.respondi.com; last access 17.01.2024). The agencies used the database information to recruit parents of children aged 8–17 years. Participants were informed of the purpose of the data collection and the privacy policy and were requested to provide consent for the assessment of sensitive data (i.e., their children’s health information). Parents were asked if their child had a history of pTBI or a serious life-threatening medical condition. If either was confirmed, participation was discontinued. All other parents were directed to the sociodemographic questions and then asked if the child was currently available. If the child was currently unavailable, the survey could be completed later. If the child was present, they were invited to participate and, after confirming readiness to begin, the pediatric PROMs were presented. Incentives were provided to participants in the form of either tokens or certificates.

**Fig. 1:**
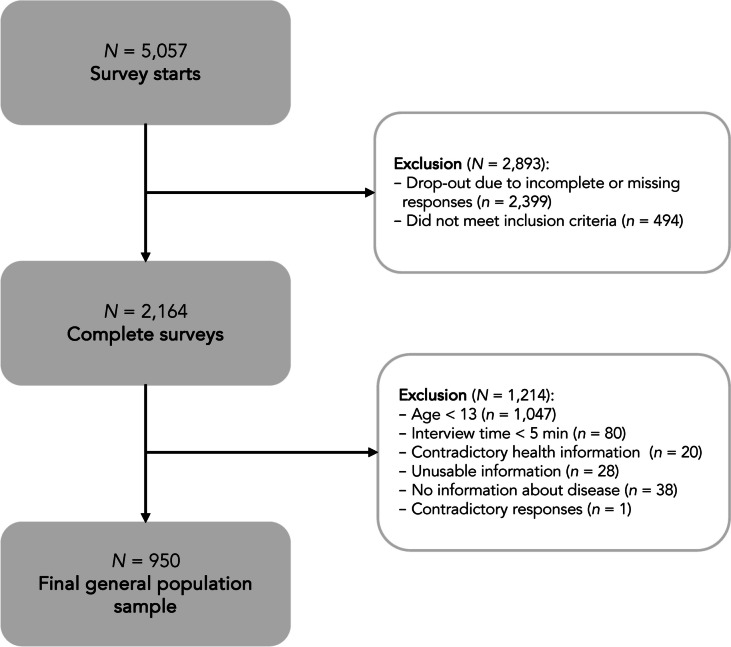
Composition of the general population sample

For data quality purposes, we excluded participants who provided inconsistent responses (e.g., reported no health problems but provided a description of their health status in the text box), unusable information (e.g., a comment not related to the question), and those who completed the survey in less than five minutes. As the survey did not allow missing responses, no further missing data were generated. The only exception was the question on receiving integration assistance at school, which was only asked if children and adolescents were attending any type of educational institution. A total of 950 adolescents aged 13–17 years from 2164 completed child and adolescent surveys were included in the analyses (see Fig. 1).

For comparative analyses on construct validity, we used PCSI-SR13 data from the pTBI population assessed in the Quality of Life after Brain Injury in Children and Adolescents project, collected in Germany and Austria from January 2019 to February 2023. The questionnaire was administered in both phases of the study, the pilot and the final validation study, with post-pTBI participants completing the PCSI-SR13 pre-post form in the first phase and the post form only in the second phase. To avoid a potential effect of repeated data assessment using the same questionnaire, data from the first phase of participation was preferred for those who participated twice. Further details on the study description, inclusion and exclusion criteria, and recruitment procedures can be found elsewhere [14]. A total of *N* = 234 adolescents after pTBI were included in the comparative analyses (see Supplementary Figure S1).

### Materials and measures

Sociodemographic data (gender, age, education level) and health-related information were reported by the parents. Questions on health status allowed multiple responses in the following nine categories: central nervous system disease; alcohol and/or psychotropic substance abuse; active or uncontrolled systemic disease; psychiatric disorders; severe sensory deficits; use of psychotropic or other medications; intellectual disability or other neurobehavioral disorder; pre-, peri-, and postnatal problems; other. If there was at least one endorsement, the presence of at least one chronic health condition was assumed.

The PCSI-SR13 [11] is a 21-item self-report questionnaire for adolescents aged 13 to 18 years covering four groups of symptoms (i.e., physical, emotional, cognitive, and fatigue). The response scale is a seven-point Guttman scale with three anchor categories: 0 (not a problem), 3 (a moderate problem), and 6 (a severe problem). For the present study, the post version of the PCSI-SR13 was administered and adapted for use in the general pediatric population by omitting the reference to pTBI. The adaptation consisted only of changing the instructions, which were worded as follows: “We would like to know if you have any of these complaints at the present time (yesterday and today).”

### Statistical analyses

To ensure the applicability of the PCSI-SR13 in the general population, we conducted psychometric analyses using the classical test theory framework. We examined the internal consistency of the instrument using Cronbach’s *α* and McDonald’s *ω* (values ≥ 0.70 desirable [15]), and calculated Cronbach’s *α* when omitting each item of the respective scale and (corrected) item-total correlations ([C]ITC; values of 0.40 or greater desirable) to further investigate the reliability.

Confirmatory factor analysis (CFA) using the diagonally weighted least square estimator for ordinal data was used to assess construct validity. Multiple goodness-of-fit indices were considered to assess model fit: ${\chi }^{2}$-value (*p* > 0.05) and ${\chi }^{2}$-value and degrees of freedom ratio (${\chi }^{2}$/*df* ≤ 2) [16], comparative fit index (CFI ≥ 0.95) [17], Tucker-Lewis index (TLI ≥ 0.95) [17], root mean square error of approximation including 90% confidence interval (CI_90%_) (excellent to close fit: RMSEA < 0.05) [18], and standardized root mean square residual (SRMR < 0.08) [17]. Scaled values were reported for all fit indices except SRMR.

To ensure that the PCSI-SR13 measures the same construct in the general population as it does in the pTBI population, we applied a measurement invariance (MI) approach involving three steps of estimating models with increasing constraints [19, 20]. Negative binomial regression models were used to examine the potential effect of gender, age, and health status and their interactions (e.g., gender × age or gender × health status) on PCSI-SR13 total and scale scores. Significant interactions indicated the need for further stratification of the reference values. Information on these analyses is provided in the Supplemental Material.

We used percentiles to provide reference values because they are the easiest to interpret in a clinical context. A percentile is a value below which a certain relative proportion of the reference population falls. The distribution of PCSI-SR13 scores in the general population sample was divided by the following percentiles: 2.5%, 5%, 16%, 30%, 40%, 50%, 60%, 70%, 85%, 95%, and 97.5%. Values exceeding 50% (i.e., the median or also the mean of the percentile distribution) by one standard deviation, which is the 85% percentile (rounded up to the next integer) for normally distributed data, were considered clinically relevant.

Finally, we performed scale-wise comparisons of total and scale scores (Mann–Whitney *U* tests) between participants from the general population sample with and without chronic health conditions separately and the pTBI sample. Propensity score-matching was used to adjust for age and gender. For comparisons between the healthy general population and the pTBI sample, matching was performed using the pTBI sample as the reference. For analyses between adolescents with chronic health conditions and the pTBI sample, matching was done the other way round, using the general population sub-sample as the reference. Only male and female participants were included due to the small size of the diverse gender group. Vargha and Delaney’s effect size (*A*) [21] was calculated and interpreted using the following classification: group equal (0.50), small (0.35–0.44 or 0.56–0.63), medium (0.30–0.34 or 0.64–0.70), and large effect (greater than 0.29 or 0.71).

All analyses were performed with R (version 4.2.3) [22] using the packages table1 [23] for descriptive statistics, *psych* [24] for psychometric analyses, *lavaan* [25] for the CFA and the MI analyses, *matchIt* for sample matching [26], and *effsize* for sample comparisons [27]. The significance level was set at *α* = 5%. Where appropriate, Bonferroni correction was applied for the number of scales (*α* = 5%/4 = 1.25%).

## Results

### Sample characteristics

The general population sample consisted of *N* = 950 adolescents (50.9% female) with a mean age of 15 ± 1.39 years. Most were attending either preparatory high school (42%) or secondary/middle school (36.1%) with no integration assistance (86.2%). According to parental reports, the majority of the adolescents did not suffer from any chronic health conditions (86.2%). For details on sample characteristics, see Table 1.

**Table 1:** Sociodemographic and health-related data of the sample

	Adolescents
	*N* = 950 (100%)
Gender	
Female	484 (50.9%)
Male	465 (48.9%)
Diverse	1 (0.1%)
Age (years)	
*M* (*SD*)	15.0 (1.39)
*Md* (*Min*, *Max*)	15.0 (13.0, 17.0)
Education	
None	6 (0.6%)
Not identified*	15 (1.6%)
Special school	36 (3.8%)
Secondary school	73 (7.7%)
Secondary school/middle school	343 (36.1%)
Vocational school	78 (8.2%)
Preparatory high school	399 (42.0%)
Integration assistance**	
Yes	125 (13.2%)
No	819 (86.2%)
Missing	6 (0.6%)
Number of chronic health conditions***	
One and more	127 (13.4%)
None	823 (86.6%)

^*^Due to implausible data, the category “not identified” was added

^**^The question on integration assistance was only presented if children and adolescents were attending any kind of educational institution

^***^The assessment of chronic conditions is based on parental report and is considered present if at least one of the following health problems is reported: central nervous system disease, alcohol and/or psychotropic substance abuse, active or uncontrolled systemic disease, psychiatric disorders, severe sensory deficits, use of psychotropic drugs or other medications, intellectual disability, or other neurobehavioral disorder, pre-, peri-, and postnatal problems, and/or other. *M* mean, *SD* standard deviation, *Md* median, *Min* minimum, *Max* maximum, *N* sample size

### Response patterns

Analysis of the response patterns per item revealed that most participants endorsed the “not a problem” category, indicating that they were not affected by the symptoms. The proportion ranged from 48% (drowsiness) to 79% (vision problems). However, when summarizing the response categories that capture at least a moderate problem (i.e., response categories 3–6), we observed a notable number of individuals reporting at least moderate headache, irritability, and drowsiness (approximately 22% each). For more details, see Supplemental Table S1.

### Psychometric properties

Table 2 provides an overview of reliability analysis results. The values of Cronbach’s *α* and McDonald’s *ω* exceeded the cut-off of 0.70 at both the scale and the total score level. None of the items contributed to the increase of the initial Cronbach’s *α* of the scale and the (C)ITCs were above 0.40.

**Table 2:** Results of reliability analyses

Scale	Item	Cronbach’s *α*ª	McDonald’s *ω*	Cronbach’s *α* if item omittedª	ITCª	CITC
Physical	Headache	**0.93**	**0.94**	**0.94**	**0.72**	**0.63**
	Nausea			**0.92**	**0.83**	**0.78**
	Balance problems			**0.92**	**0.87**	**0.82**
	Dizziness			**0.92**	**0.87**	**0.82**
	Visual problems (double vision, blurring)			**0.93**	**0.81**	**0.74**
	Move in a clumsy manner			**0.93**	**0.81**	**0.75**
	Sensitivity to light			**0.92**	**0.86**	**0.82**
	Sensitivity to noise			**0.92**	**0.84**	**0.78**
Emotional	Irritability	**0.91**	**0.92**	**0.89**	**0.87**	**0.76**
	Sadness			**0.88**	**0.89**	**0.8**
	Nervousness			**0.88**	**0.88**	**0.78**
	Feeling more emotional			**0.87**	**0.90**	**0.81**
Cognitive	Feeling mentally foggy	**0.94**	**0.95**	**0.94**	**0.84**	**0.76**
	Difficulty concentrating			**0.94**	**0.85**	**0.78**
	Difficulty remembering			**0.93**	**0.89**	**0.84**
	Get confused with directions or tasks			**0.93**	**0.88**	**0.83**
	Answer questions more slowly than usual			**0.92**	**0.91**	**0.87**
	Feeling slowed down			**0.93**	**0.90**	**0.84**
Fatigue	Fatigue	**0.90**	**0.90**	**0.86**	**0.90**	**0.78**
	Drowsiness			**0.82**	**0.93**	**0.84**
	Sleeping more than usual			**0.87**	**0.90**	**0.77**
Total		**0.97**	**0.97**	-	-	-

ªStandardized *α* coefficients are used. *(C)ITC* (corrected) item-total correlations. Values in bold are within acceptable cut-offs (i.e., *α* and *ω* greater than or equal to 0.70, *α* if an item omitted do not exceeding the initial α of the scale and [C]ITC greater than or equal to 0.40)

The four-factor structure could be replicated in the general population sample with almost all goodness-of-fit indices not exceeding permissible cut-offs: *χ*^2^(183) = 995.96, *p* < 0.001, *χ*^2^/*df* = 5.44, CFI = 0.99, TLI = 0.98, RMSEA (90% CI) = 0.068 (0.064, 0.073), SRMR = 0.03.

### Reference values

The comparability of the construct assessment between general population and pTBI samples and the factors influencing the PCSI-SR13 total and scale scores in the general population sample were examined prior to providing reference values. We found no substantial differences in the models with increasing constraints that would cause the PCS construct assessment to deviate between the two samples (see Supplementary Table S3). Furthermore, no significant interactions were found between gender, age, and presence of chronic health conditions on PCSI-SR13 total and scale scores (see Supplementary Table S4). Therefore, we considered the PCS assessment to be comparable between the pTBI and general population samples and provided unstratified reference values for further use in clinical practice. To avoid potential bias and to provide reference values from the general population with no history of chronic health conditions, we excluded *N* = 127 adolescents who reported any chronic complaints.

The reference values in Table 3 provide a basis for clinical screening decisions when using the post version of the PCSI-SR13 in adolescents aged 13 to 17 years. Here is an example of how to use these reference values: Suppose an adolescent has a PCSI-SR13 score of 38 after a pTBI. Compared to a general population sample, his or her score falls between the 85th and 95th percentiles. The score can be considered clinically relevant because it is above average and more than 85% of the general population has a lower score. The PCSI-SR13 scale scores can be treated in a similar manner. Therefore, a specific symptom domain (e.g., emotional or physical) can be screened for clinical relevance to identify potential problem areas. Alternatively, the following cut-off values can be used: values of 36 (total score), 12 (physical), 10 (emotional), 10 (cognitive), and 8 (fatigue) can be used to classify patient-reported scores as not clinically relevant (i.e., equal to or below the respective cut-offs) and clinically relevant (i.e., above the respective cut-offs). Reference values are also available in an interactive application at https://reference-values.shinyapps.io/Tables_Reference_values/ (tab PCSI-SR13; last access 17.01.2024).

**Table 3:** Reference values

		Low symptoms severity		− 1 *SD*			*Md*			+ 1 *SD*		High symptoms severity
Scale	*N*	2.5%	5%	16%	30%	40%	50%	60%	70%	85%	95%	97.5%
Total	823	0	0	0	2	5	7	11	17	36	72	83
Physical		0	0	0	0	0	1	3	4	12	26	32
Emotional		0	0	0	0	1	2	3	5	10	15	18
Cognitive		0	0	0	0	0	1	2	4	10	21	25
Fatigue		0	0	0	0	0	1	2	4	8	12	14

*N* = 127 adolescents with chronic health conditions were not included in the reference values. 50% percentiles represent 50% of the distribution corresponding to the median (*Md*) and the mean of the distribution; *SD* standard deviation; values from − 1 standard deviation (16%, rounded up to the next integer) to + 1 standard deviation (85%, rounded up to the next integer) are within the normal range (i.e., not clinically relevant symptom severity); values below 16% indicate low symptoms severity (i.e., absence of PCSI-SR-13 symptoms) and values above 85% indicate high symptom severity (i.e., presence of clinically relevant PCSI-SR13 symptoms)

### Score differences

Matching resulted in *N* = 233 dyads from the general population without chronic health conditions and the pTBI sample, with a propensity score variance ratio of 1 for perfect alignment. Comparisons revealed significant differences between the samples on the scales and in the total score, with children and adolescents after pTBI reporting more intense symptoms (*p* < 0.001). The effect sizes were small (fatigue scale) to medium (total score). For details, see Table 4 (left part).

**Table 4:** Comparisons between the matched general population sample (healthy and with at least one chronic health condition) and the pTBI sample on the PCSI-SR13 total score and the PCSI-SR13 scale scores

Scale	Characteristic	Healthy general population sample vs. pTBI sample					General population sample with at least one chronic health condition vs. pTBI sample				
		General population (*N* = 233)	pTBI (*N* = 233)	*U*	*p*	*A*	General population (*N* = 126)	pTBI (*N* = 126)	*U*	*p*	*A*
Total	*M* (*SD*)	13.4 (20.2)	20.3 (18.4)	17,566	**< 0.001**	0.32	27.7 (23.6)	21.1 (19.0)	6764	**0.042**	0.57
	*Md* (*Min*, *Max*)	5.00 (0, 106)	14.0 (0, 82.0)				20.5 (0, 105)	15.0 (0, 82.0)			
Physical	*M* (*SD*)	3.89 (7.39)	6.32 (6.92)	17,666	**< 0.001**	0.33	7.50 (8.59)	6.26 (6.57)	7729.5	0.718	0.51
	*Md* (*Min*, *Max*)	1.00 (0, 47.0)	4.00 (0, 35.0)				4.50 (0, 42.0)	4.00 (0, 35.0)			
Emotional	*M* (*SD*)	3.28 (4.67)	4.93 (5.08)	19,583	**< 0.001**	0.36	7.18 (6.14)	5.23 (5.48)	6456	**0.01**	0.59
	*Md* (*Min*, *Max*)	1.00 (0, 21.0)	3.00 (0, 24.0)				6.00 (0, 24.0)	3.00 (0, 24.0)			
Cognitive	*M* (*SD*)	3.54 (6.32)	5.48 (5.70)	18,060	**< 0.001**	0.33	7.87 (8.39)	6.04 (6.02)	7492	0.439	0.53
	*Md* (*Min*, *Max*)	1.00 (0, 33.0)	4.00 (0, 25.0)				5.00 (0, 35.0)	4.50 (0, 25.0)			
Fatigue	*M* (*SD*)	2.67 (3.76)	3.52 (3.72)	21,496	**< 0.001**	0.40	5.10 (4.61)	3.60 (3.92)	6468.5	**0.01**	0.59
	*Md* (*Min*, *Max*)	1.00 (0, 15.0)	3.00 (0, 17.0)				4.00 (0, 18.0)	3.00 (0, 17.0)			

*M* mean, *SD* standard deviation, *Md* median, *Min* minimum, *Max* maximum, *U* Mann–Whitney *U* test statistic, *p p*-value, *A* Vargha and Delaney’s effect size: group equal (0.50), small (0.35–0.44 or 0.56–0.63), medium (0.30–0.34 or 0.64–0.70), and large effect (greater than 0.29 or 0.71). Values in bold are significant at 5% (total) or 1.25% (scale scores)

Matching participants from the general population sample with at least one chronic health condition to the pTBI sample (*N* = 126 dyads) resulted in a propensity score variance ratio of 0.99, indicating near-perfect alignment. The samples differed significantly on the total score (*p* = 0.042), with the emotional and fatigue scales contributing to this difference. In all cases, adolescents from the general population sample reported significantly more intense symptoms than those from the pTBI sample, although the effect sizes were small (Table 4, right part).

## Discussion

The aim of the present study was to provide reference values from the German general pediatric population for the post version of the PCSI-SR13 to assess symptom burden after pTBI. Assessment of PCS can be challenging, especially in adolescents who may present with similar symptoms due to puberty (e.g., headaches and other pains [28], concentration and remembering difficulties [29], or fatigue [30]). Therefore, it is important to provide reference values obtained from a comparable general pediatric population. With these values, clinicians and researchers can now perform time-efficient screening of PCS to tailor diagnostic and therapeutic approaches in pTBI patients.

Adolescents from the general population sample reported a notable number and severity of post-concussion-like symptoms. A similar finding has already been reported for adult populations in three European countries (Italy, the Netherlands, and the UK) [31]. In the present study, comparisons between the general population sub-sample suffering from at least one chronic health condition and the pTBI sample show significant differences in emotional distress and fatigue symptoms in favor of the non-pTBI adolescents. It is therefore particularly important to differentiate between symptoms caused by pTBI and those that were present prior to injury for other reasons (e.g., symptoms due to maturation or chronic health conditions such as systemic diseases or neurobehavioral disorders) in order to ensure appropriate diagnosis. In the current sample, headache, irritability, and drowsiness were the most common self-reported symptoms, which is in line with previous findings [32, 33]. These symptoms may be due to the developmental stage of the participating adolescents (e.g., changes in sex hormones, brain structure and function, or psychological/social changes [28]), the presence of health conditions other than pTBI [34], and the cross-sectional study design, which is likely to have neglected variability in symptom burden [35]. Overall, researchers agree that PCS (post-concussion symptoms) after pTBI tend to be injury non-specific, have a high overlap with other health conditions (e.g., depression), and should therefore be evaluated with caution [34]. In particular, an appropriate review of the medical history and anamnesis can facilitate diagnostic decisions [34]. A further comparison with the general population, as provided in the present study, would additionally allow differentiation of the extent of pTBI-related symptoms. Considering that the direct comparison of PCSI-SR13 scores indicates a significantly higher symptom burden in pTBI patients compared to age- and gender-matched general population sample, the provided reference values can be used for PCS screening, especially when pTBI patients do not report any chronic health conditions prior to injury.

### Strengths and limitations

The main advantage of the present study is that it fills the gap of reference values for the German version of the post version of the age-adapted PCSI-SR13. The reference values provided are derived from a large general population sample and may be particularly helpful when time is of the essence and a quick screening decision is needed. However, the study has also some potential limitations. Although online surveys can reach large numbers of participants in a relatively short period of time, they can be subject to bias [36]. We have tried to minimize the impact of potential bias by partnering with recognized international research organizations and providing quality control of the data. Data from adolescents with chronic health conditions have been excluded from the reference values. Therefore, the reference values represent the ideal norm. Supplementing the reference values with information from populations with chronic health conditions would provide additional understanding of post-concussion-like symptoms in the general population and enable more differentiated comparisons with pTBI patients.

## Conclusions

The present study established reference values for the German version of the PCSI-SR13 to assess PCS burden in an adolescent pTBI population. This advancement allows clinicians and researchers to conduct time-efficient screenings while taking into account the prevalence and intensity of post-concussion-like symptoms in the healthy general population, thereby facilitating more accurate diagnoses and tailored clinical diagnostic and therapeutic strategies.

## Supplementary Information

Below is the link to the electronic supplementary material.

Supplementary file1 (DOCX 8701 KB)

## Data Availability

The datasets analyzed during the current study are not publicly available due to data protection policy, but are available from the corresponding author upon reasonable request.

## Abbreviations

CFA: Confirmatory factor analysis
CFI: Comparative fit index
CI: Confidence interval
(C)ITC: (Corrected) item-total correlations
MI: Measurement invariance
PCS: Post-concussion symptoms
PCSI: Postconcussion Symptom Inventory
PROM: Patient-reported outcome measure
pTBI: Pediatric traumatic brain injury
RMSEA: Root mean square error of approximation
SRMR: Standardized root mean square residual
TLI: Tucker-Lewis index
