# Supplementary material for: Reference values for the adolescent post version of the Postconcussion Symptom Inventory from the German general population
Source: Eur J Pediatr. 2024 Dec 23;184(1):103. doi: 10.1007/s00431-024-05906-8 (PMC11666766; doi:10.1007/s00431-024-05906-8)
Supplement: Supplementary file 1 — Supplementary file1 (DOCX 8701 KB) [file 431_2024_5906_MOESM1_ESM.docx]

# Supplemental Material

*Participants after pTBI*

**Supplemental Figure S1** Composition of the TBI sample (*N* = 234). First study phase (light grey rectangles): pilot study of the Quality of Life after Brain Injury in Children and Adolescents project [1]; second study phase (dark grey rectangles): final validation study of the Quality of Life after Brain Injury in Children and Adolescents project [2].

**Supplemental Table S1** Response patterns of the general population sample (*N* = 950).

|  |  | Response patterns (*n*, %) | | | | | | |  |
| --- | --- | --- | --- | --- | --- | --- | --- | --- | --- |
| **Scale** | **Item** | **0 Not a problem** | **1** | **2** | **3 Moderate problem** | **4** | **5** | **6 Severe problem** | **At least a moderate problem**  **(3–6)** |
| **Physical** | Headache | 544 (57.3%) | 107 (11.3%) | 88 (9.3%) | 114 (12.0%) | 51 (5.4%) | 28 (2.9%) | 18 (1.9%) | 211 (22.2%) |
|  | Nausea | 706 (74.3%) | 89 (9.4%) | 36 (3.8%) | 56 (5.9%) | 35 (3.7%) | 22 (2.3%) | 6 (0.6%) | 119 (12.5%) |
|  | Balance problems | 750 (78.9%) | 61 (6.4%) | 42 (4.4%) | 54 (5.7%) | 26 (2.7%) | 9 (0.9%) | 8 (0.8%) | 97 (10.2%) |
|  | Dizziness | 722 (76.0%) | 69 (7.3%) | 51 (5.4%) | 54 (5.7%) | 28 (2.9%) | 16 (1.7%) | 10 (1.1%) | 108 (11.4%) |
|  | Visual problems (double vision, blurring) | 753 (79.3%) | 68 (7.2%) | 43 (4.5%) | 46 (4.8%) | 17 (1.8%) | 16 (1.7%) | 7 (0.7%) | 86 (9.1%) |
|  | Move in a clumsy manner | 638 (67.2%) | 132 (13.9%) | 73 (7.7%) | 60 (6.3%) | 22 (2.3%) | 20 (2.1%) | 5 (0.5%) | 107 (11.3%) |
|  | Sensitivity to light | 722 (76.0%) | 76 (8.0%) | 46 (4.8%) | 42 (4.4%) | 33 (3.5%) | 17 (1.8%) | 14 (1.5%) | 106 (11.2%) |
|  | Sensitivity to noise | 669 (70.4%) | 94 (9.9%) | 65 (6.8%) | 50 (5.3%) | 33 (3.5%) | 21 (2.2%) | 18 (1.9%) | 122 (12.8%) |
| **Emotional** | Irritability | 477 (50.2%) | 139 (14.6%) | 122 (12.8%) | 102 (10.7%) | 56 (5.9%) | 32 (3.4%) | 22 (2.3%) | 212 (22.3%) |
|  | Sadness | 527 (55.5%) | 166 (17.5%) | 83 (8.7%) | 88 (9.3%) | 42 (4.4%) | 26 (2.7%) | 18 (1.9%) | 174 (18.3%) |
|  | Nervousness | 554 (58.3%) | 140 (14.7%) | 105 (11.1%) | 73 (7.7%) | 45 (4.7%) | 26 (2.7%) | 7 (0.7%) | 151 (15.9%) |
|  | Feeling more emotional | 557 (58.6%) | 133 (14.0%) | 79 (8.3%) | 92 (9.7%) | 46 (4.8%) | 30 (3.2%) | 13 (1.4%) | 181 (19.1%) |
| **Cognitive** | Feeling mentally foggy | 735 (77.4%) | 79 (8.3%) | 42 (4.4%) | 42 (4.4%) | 29 (3.1%) | 13 (1.4%) | 10 (1.1%) | 94 (9.9%) |
|  | Difficulty concentrating | 483 (50.8%) | 175 (18.4%) | 108 (11.4%) | 86 (9.1%) | 45 (4.7%) | 39 (4.1%) | 14 (1.5%) | 184 (19.4%) |
|  | Difficulty remembering | 603 (63.5%) | 135 (14.2%) | 81 (8.5%) | 50 (5.3%) | 47 (4.9%) | 26 (2.7%) | 8 (0.8%) | 131 (13.8%) |
|  | Get confused with directions or tasks | 589 (62.0%) | 130 (13.7%) | 76 (8.0%) | 77 (8.1%) | 44 (4.6%) | 27 (2.8%) | 7 (0.7%) | 155 (16.3%) |
|  | Answer questions more slowly than usual | 678 (71.4%) | 99 (10.4%) | 54 (5.7%) | 62 (6.5%) | 28 (2.9%) | 20 (2.1%) | 9 (0.9%) | 119 (12.5%) |
|  | Feeling slowed down | 708 (74.5%) | 95 (10.0%) | 38 (4.0%) | 54 (5.7%) | 23 (2.4%) | 27 (2.8%) | 5 (0.5%) | 109 (11.5%) |
| **Fatigue** | Fatigue | 509 (53.6%) | 137 (14.4%) | 112 (11.8%) | 99 (10.4%) | 48 (5.1%) | 33 (3.5%) | 12 (1.3%) | 192 (20.2%) |
|  | Drowsiness | 460 (48.4%) | 167 (17.6%) | 105 (11.1%) | 118 (12.4%) | 44 (4.6%) | 39 (4.1%) | 17 (1.8%) | 218 (22.9%) |
|  | Sleeping more than usual | 593 (62.4%) | 124 (13.1%) | 63 (6.6%) | 77 (8.1%) | 53 (5.6%) | 22 (2.3%) | 18 (1.9%) | 170 (17.9%) |
| *Note*. *n* = absolute frequencies, % = relative frequencies. | | | | | | | | | |

## *Measurement invariance and regression analyses*

For the measurement invariance (MI) analyses, the first step was to estimate a baseline model. Then, the equality of thresholds across groups was tested. Finally, thresholds and loadings were expected to be equal in both samples. Goodness of fit was assessed using the same indices as in the CFA analysis, and models were compared using the difference test, expecting insignificant results (p ≥ 0.05). In addition to the p-value, differences in CFI and RMSEA were considered, with ΔCFI < 0.01 [3] and ΔRMSEA ≤ 0.01 [4], respectively, suggesting no meaningful differences between the models. Visualization of parameter differences between the general population and the TBI sample in the models with different constraints additionally served as an indication of (non-)violation of the MI, with differences not exceeding 5% considered negligible. For the TBI sample, to preserve information, missing item values were replaced with the corresponding scale means if at least two-thirds of the items on the scale were valid. This resulted in a total of 13 missing item values being replaced.

Prior to the analyses, the response patterns of the TBI sample were examined (see Supplementary Table S2). Response pattern analysis revealed that TBI sample participants most frequently rated headache, irritability, feeling of mental fogginess, fatigue, and drowsiness as at least a moderate problem (approximately 24% to 27% of the sample). In general, higher response categories indicating greater symptom burden (i.e., 4–6) were rarely selected (0% to 10%). In particular, participants did not use all response categories on the items measuring nausea, dizziness, clumsiness, sensitivity to noise, and feeling of mental fogginess; on these items, the highest response category “severe problem” was not endorsed (0%). Therefore, to perform the MI analyses, we collapsed the responses according to the classification used for descriptive analyses in the English PCSI validation study (0: 0, 1: 1–3, 2: 4–6) [5].

All three models fit the data well according to the model fit statistics (see Supplementary Table S3). Due to the equal model parameters, no comparisons were made between the baseline and threshold models. A significant difference (p < 0.001) was observed between the thresholds and the thresholds and loading model. However, ΔCFI < 0.001 and ΔRMSEA = 0.001 did not exceed respective cut-offs, indicating that the differences were negligible. To better understand and explain the differences found, we provided a visualization of the model parameters (Supplementary Figure S2). For this purpose, the differences in threshold parameters between the general population and TBI samples in the threshold model were plotted against the differences in thresholds and thresholds and loadings model for each item. None of the differences exceeded 5%. Therefore, the construct assessment was considered to be largely comparable between the two samples, and further steps were followed to provide reference values.

Regression analyses revealed no significant interactions for either the total score or the scale scores (see Supplementary Table S4). Therefore, no further stratification of reference values was performed.

**Supplemental Table S2** Response patterns of the TBI sample (*N* = 234).

|  |  | Response patterns (*n*, %) | | | | | | | |
| --- | --- | --- | --- | --- | --- | --- | --- | --- | --- |
| **Scale** | **Item** | **0 Not a problem** | **1** | **2** | **3 Moderate problem** | **4** | **5** | **6 Severe problem** | **At least a moderate problem**  **(3–6)** |
| **Physical** | Headache | 107 (45.7%) | 42 (17.9%) | 26 (11.1%) | 31 (13.2%) | 18 (7.7%) | 7 (3.0%) | 3 (1.3%) | 59 (25.2%) |
|  | Nausea | 163 (69.7%) | 37 (15.8%) | 17 (7.3%) | 8 (3.4%) | 7 (3.0%) | 2 (0.9%) | **0 (0.0%)** | 17 (7.3%) |
|  | Balance problems | 152 (65.0%) | 35 (15.0%) | 23 (9.8%) | 14 (6.0%) | 6 (2.6%) | 2 (0.9%) | 2 (0.9%) | 24 (10.3%) |
|  | Dizziness | 137 (58.5%) | 42 (17.9%) | 20 (8.5%) | 23 (9.8%) | 5 (2.1%) | 7 (3.0%) | **0 (0.0%)** | 35 (15.0%) |
|  | Visual problems (double vision, blurring) | 163 (69.7%) | 34 (14.5%) | 14 (6.0%) | 11 (4.7%) | 6 (2.6%) | 2 (0.9%) | 4 (1.7%) | 23 (9.8%) |
|  | Move in a clumsy manner | 129 (55.1%) | 63 (26.9%) | 19 (8.1%) | 15 (6.4%) | 5 (2.1%) | 3 (1.3%) | **0 (0.0%)** | 23 (9.8%) |
|  | Sensitivity to light | 170 (72.6%) | 27 (11.5%) | 20 (8.5%) | 12 (5.1%) | 1 (0.4%) | 3 (1.3%) | 1 (0.4%) | 17 (7.3%) |
|  | Sensitivity to noise | 144 (61.5%) | 32 (13.7%) | 26 (11.1%) | 18 (7.7%) | 11 (4.7%) | 3 (1.3%) | **0 (0.0%)** | 32 (13.7%) |
| **Emotional** | Irritability | 79 (33.8%) | 60 (25.6%) | 35 (15.0%) | 34 (14.5%) | 14 (6.0%) | 9 (3.8%) | 3 (1.3%) | 60 (25.6%) |
|  | Sadness | 113 (48.3%) | 45 (19.2%) | 31 (13.2%) | 18 (7.7%) | 14 (6.0%) | 9 (3.8%) | 4 (1.7%) | 45 (19.2%) |
|  | Nervousness | 109 (46.6%) | 48 (20.5%) | 31 (13.2%) | 27 (11.5%) | 10 (4.3%) | 5 (2.1%) | 4 (1.7%) | 46 (19.7%) |
|  | Feeling more emotional | 134 (57.3%) | 38 (16.2%) | 22 (9.4%) | 16 (6.8%) | 14 (6.0%) | 9 (3.8%) | 1 (0.4%) | 40 (17.1%) |
| **Cognitive** | Feeling mentally foggy | 160 (68.4%) | 28 (12.0%) | 23 (9.8%) | 17 (7.3%) | 5 (2.1%) | 1 (0.4%) | **0 (0.0%)** | 23 (9.8%) |
|  | Difficulty concentrating | 78 (33.3%) | 59 (25.2%) | 35 (15.0%) | 29 (12.4%) | 23 (9.8%) | 8 (3.4%) | 2 (0.9%) | 62 (26.5%) |
|  | Difficulty remembering | 105 (44.9%) | 62 (26.5%) | 28 (12.0%) | 16 (6.8%) | 13 (5.6%) | 6 (2.6%) | 4 (1.7%) | 39 (16.7%) |
|  | Get confused with directions or tasks | 102 (43.6%) | 63 (26.9%) | 36 (15.4%) | 19 (8.1%) | 10 (4.3%) | 2 (0.9%) | 2 (0.9%) | 33 (14.1%) |
|  | Answer questions more slowly than usual | 161 (68.8%) | 39 (16.7%) | 17 (7.3%) | 9 (3.8%) | 5 (2.1%) | 2 (0.9%) | 1 (0.4%) | 17 (7.3%) |
|  | Feeling slowed down | 172 (73.5%) | 37 (15.8%) | 12 (5.1%) | 5 (2.1%) | 4 (1.7%) | 3 (1.3%) | 1 (0.4%) | 13 (5.6%) |
| **Fatigue** | Fatigue | 87 (37.2%) | 61 (26.1%) | 26 (11.1%) | 35 (15.0%) | 16 (6.8%) | 8 (3.4%) | 1 (0.4%) | 60 (25.6%) |
|  | Drowsiness | 93 (39.7%) | 57 (24.4%) | 28 (12.0%) | 29 (12.4%) | 16 (6.8%) | 7 (3.0%) | 4 (1.7%) | 56 (23.9%) |
|  | Sleeping more than usual | 158 (67.5%) | 23 (9.8%) | 28 (12.0%) | 13 (5.6%) | 8 (3.4%) | 1 (0.4%) | 3 (1.3%) | 25 (10.7%) |
| *Note*. *n* = absolute frequencies, % = relative frequencies. Values in **bold** indicate frequencies n = 0. | | | | | | | | | |

**Supplementary Table S3** Results of measurement invariance analyses.

| Samples | Constraints | *χ*2 (*df*) | *p* | *χ*2/*df* | CFI | TLI | RMSEA [90% CI] | SRMR | Δ*χ*2 | Δ*df* | *p* | ΔCFI | ΔRMSEA |
| --- | --- | --- | --- | --- | --- | --- | --- | --- | --- | --- | --- | --- | --- |
| General population sample  vs.  pTBI sample | baseline | 855.89 (366) | < 0.001 | 2.34 | **0.99** | **0.99** | **0.048 [0.043, 0.052]** | **0.04** | - | - | - | - | - |
|  | thresholds | 855.89 (366) | < 0.001 | 2.34 | **0.99** | **0.99** | **0.048 [0.043, 0.052]** | **0.04** | - | - | - | - | - |
|  | thresholds and loadings | 888.95 (383) | <0.001 | 2.32 | **0.99** | **0.99** | **0.047 [0.043, 0.051]** | **0.04** | 48.39 | 17 | < 0.001 | **<0.001** | **0.001** |
| *Note*. χ2: scaled chi-square statistics; df: scaled degrees of freedom; *p: p*-value; χ2/df: scaled ratio (cut-off: ≤ 2); CFI: scaled Comparative Fit Index (cut-off: > 0.90); TLI: scaled Tucker-Lewis Index (cut-off: > 0.95); RMSEA [90%CI]: scaled root mean square error of approximation with 90% confidence interval (cut-off: < 0.06); SRMR: scaled Standardized Root Mean Square Residual (cut-off: < 0.08); Δ*χ*2: chi-square statistics of the difference test; Δ*df* : degrees of freedom of the difference test. The baseline model and the threshold model have the same test statistics, therefore a test for difference is not performed. Values in **bold** indicate at least satisfactory/mediocre model fit according to the respective cut-offs and/or are within acceptable range. | | | | | | | | | | | | | |


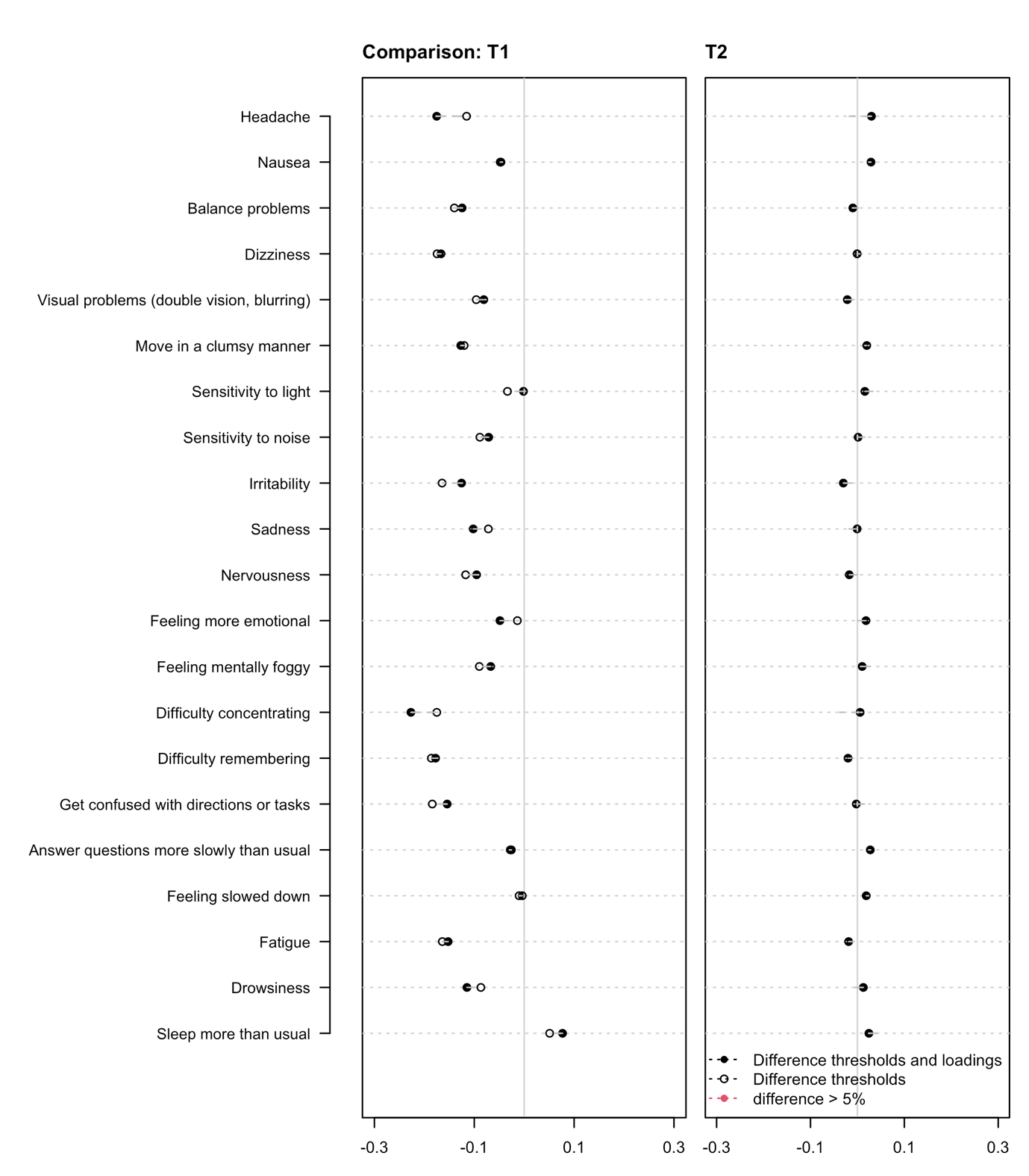


**Supplementary Figure S2** Differences in the threshold parameters between the general population and pTBI samples in the threshold model (unfilled circles) were plotted against the differences in thresholds and thresholds and loadings model (filled circles) for each item with a trichotomized response scale (0: 0, 1: 1–3, 2: 4–6).

**Supplementary Table S4** Results of the negative binomial regression analyses for the general population sample (N = 950).

| Scale | Variable | Reference Group | Estimate | S.E. | *z* | *p* |
| --- | --- | --- | --- | --- | --- | --- |
| Total score | Intercept | - | 4.15 | 1.50 | 2.78 | **0.006** |
|  | Age in years | - | -0.04 | 0.10 | -0.45 | 0.657 |
|  | Gender | Female | 0.92 | 1.05 | 0.87 | 0.385 |
|  | Presence of chronic health conditions | At least one chronic health condition | -0.78 | 1.53 | -0.51 | 0.611 |
|  | Age in years*Gender | Age in years*Female | -0.09 | 0.07 | -1.28 | 0.200 |
|  | Age in years*Presence of chronic health conditions | Age in years*At least one chronic health condition | 0.01 | 0.10 | 0.09 | 0.927 |
|  | Gender* Presence of chronic health conditions | Female*At least one chronic health condition | 0.27 | 0.28 | 0.96 | 0.337 |
| Physical | Intercept | - | 3.65 | 1.81 | 2.02 | 0.043 |
|  | Age in years | - | -0.10 | 0.12 | -0.81 | 0.421 |
|  | Gender | Female | 0.68 | 1.28 | 0.53 | 0.594 |
|  | Presence of chronic health conditions | At least one chronic health condition | -1.24 | 1.85 | -0.67 | 0.502 |
|  | Age in years*Gender | Age in years*Female | -0.08 | 0.08 | -0.94 | 0.347 |
|  | Age in years*Presence of chronic health conditions | Age in years*At least one chronic health condition | 0.05 | 0.12 | 0.40 | 0.688 |
|  | Gender* Presence of chronic health conditions | Female*At least one chronic health condition | 0.35 | 0.34 | 1.04 | 0.298 |
| Emotional | Intercept | - | 2.15 | 1.50 | 1.43 | 0.153 |
|  | Age in years | - | 0.00 | 0.10 | 0.05 | 0.961 |
|  | Gender | Female | 0.66 | 1.08 | 0.62 | 0.538 |
|  | Presence of chronic health conditions | At least one chronic health condition | -0.13 | 1.54 | -0.08 | 0.934 |
|  | Age in years*Gender | Age in years*Female | -0.08 | 0.07 | -1.18 | 0.238 |
|  | Age in years*Presence of chronic health conditions | Age in years*At least one chronic health condition | -0.04 | 0.10 | -0.43 | 0.667 |
|  | Gender* Presence of chronic health conditions | Female*At least one chronic health condition | 0.37 | 0.28 | 1.32 | 0.189 |
| Cognitive | Intercept | - | 3.66 | 1.80 | 2.03 | 0.042 |
|  | Age in years | - | -0.10 | 0.12 | -0.85 | 0.396 |
|  | Gender | Female | 1.03 | 1.28 | 0.80 | 0.423 |
|  | Presence of chronic health conditions | At least one chronic health condition | -1.34 | 1.84 | -0.73 | 0.468 |
|  | Age in years*Gender | Age in years*Female | -0.08 | 0.08 | -1.00 | 0.319 |
|  | Age in years*Presence of chronic health conditions | Age in years*At least one chronic health condition | 0.04 | 0.12 | 0.35 | 0.730 |
|  | Gender* Presence of chronic health conditions | Female*At least one chronic health condition | 0.23 | 0.33 | 0.68 | 0.496 |
| Fatigue | Intercept | - | 1.12 | 1.52 | 0.74 | 0.460 |
|  | Age in years | - | 0.04 | 0.10 | 0.42 | 0.673 |
|  | Gender | Female | 1.36 | 1.09 | 1.25 | 0.212 |
|  | Presence of chronic health conditions | At least one chronic health condition | -0.21 | 1.56 | -0.13 | 0.894 |
|  | Age in years*Gender | Age in years*Female | -0.11 | 0.07 | -1.54 | 0.124 |
|  | Age in years*Presence of chronic health conditions | Age in years*At least one chronic health condition | -0.02 | 0.10 | -0.21 | 0.835 |
|  | Gender* Presence of chronic health conditions | Female*At least one chronic health condition | 0.05 | 0.28 | 0.19 | 0.854 |
| *Note*. *: interaction between the variables; Estimate: regression coefficient; S.E.: standard error; *z*: *z*-value; *p*: *p*-value; values in **bold** are significant at 5% for the total score or at 1.25% for the scale scores. | | | | | | |

# References

1. Von Steinbuechel N, Zeldovich M, Greving S, et al (2023) Quality of Life after Brain Injury in Children and Adolescents (QOLIBRI-KID/ADO)—The First Disease-Specific Self-Report Questionnaire after Traumatic Brain Injury. JCM 12:4898. https://doi.org/10.3390/jcm12154898

2. von Steinbuechel N, Zeldovich M, Timmermann D, et al (2024) Final Validation of the Quality of Life after Brain Injury for Children and Adolescents (QOLIBRI-KID/ADO) Questionnaire. Children 11:. https://doi.org/10.3390/children11040438

3. Hirschfeld G, Brachel R von (2014) Improving Multiple-Group confirmatory factor analysis in R – A tutorial in measurement invariance with continuous and ordinal indicators. https://doi.org/10.7275/QAZY-2946

4. Cheung GW, Rensvold RB (2002) Evaluating goodness-of-fit indexes for testing measurement invariance. Structural equation modeling 9:233–255

5. Sady MD, Vaughan CG, Gioia GA (2014) Psychometric Characteristics of the Postconcussion Symptom Inventory in Children and Adolescents. Archives of Clinical Neuropsychology 29:348–363. https://doi.org/10.1093/arclin/acu014
